# Enhancement of elastin expression by transdermal administration of sialidase isozyme Neu2

**DOI:** 10.1038/s41598-021-82820-1

**Published:** 2021-02-08

**Authors:** Akira Minami, Yuka Fujita, Jun Goto, Ayano Iuchi, Kosei Fujita, Yasuyo Mikami, Mako Shiratori, Ami Ishii, Samir Mitragotri, Yasunori Iwao, Hiroaki Kanazawa, Yuuki Kurebayashi, Tadanobu Takahashi, Tadamune Otsubo, Kiyoshi Ikeda, Takashi Suzuki

**Affiliations:** 1 Department of Biochemistry, School of Pharmaceutical Sciences, University of Shizuoka, Shizuoka, 422-8526 Japan; 2 Laboratory of Synthetic Organic and Medicinal Chemistry, School of Pharmaceutical Sciences, University of Shizuoka, Shizuoka, 422-8526 Japan; 3 School of Engineering and Applied Sciences, Harvard University, Cambridge, MA 02138 USA; 4 Department of Functional Anatomy, School of Nursing, University of Shizuoka, Shizuoka, 422-8526 Japan; 5 Department of Organic Chemistry, School of Pharmaceutical Sciences, Hiroshima International University, Hiroshima, 737-0112 Japan

**Keywords:** Health care, Drug discovery, Drug delivery

## Abstract

Reduction of elastin in the skin causes various skin diseases as well as wrinkles and sagging with aging. Sialidase is a hydrolase that cleaves a sialic acid residue from sialoglycoconjugate. Cleavage of sialic acid from microfibrils by the sialidase isozyme Neu1 facilitates elastic fiber assembly. In the present study, we showed that a lower layer of the dermis and muscle showed relatively intense sialidase activity. The sialidase activity in the skin decreased with aging. Choline and geranate (CAGE), one of the ionic liquids, can deliver the sialidase subcutaneously while maintaining the enzymatic activity. The elastin level in the dermis was increased by applying sialidase from *Arthrobacter ureafaciens* (AUSA) with CAGE on the skin for 5 days in rats and senescence-accelerated mice prone 1 and 8. Sialidase activity in the dermis was considered to be mainly due to Neu2 based on the expression level of sialidase isozyme mRNA. Transdermal administration of Neu2 with CAGE also increased the level of elastin in the dermis. Therefore, not only Neu1 but also Neu2 would be involved in elastic fiber assembly. Transdermal administration of sialidase is expected to be useful for improvement of wrinkles and skin disorders due to the loss of elastic fibers.

Sialidase is a hydrolase that releases sialic acid from sialoglycoconjugates in oligosaccharides, glycolipids and glycoproteins. Mammalian sialidase has four isozymes, Neu1, Neu2, Neu3, and Neu4. These four isozymes have differences in the tissues they are expressed in, subcellular locations, substrate specificity, and pH dependency^1,2^. We previously developed benzothiazolylphenol-based sialic acid derivative type 3 (BTP3-Neu5Ac), which is a highly sensitive fluorescent probe for histochemical imaging of sialidase activity^3^. While staining various tissues with a BTP3-Neu5Ac, we previously revealed the distributions of sialidase activity in the brain and pancreas^3,4^. Based on this information, we have found that sialidase is involved in memory processing and the regulation of glutamate and insulin release^4,5^. In the course of staining with BTP3-Neu5Ac in mammalian tissues, we newly found that the dermis has an intense sialidase activity in the skin.

Skin aging is closely associated with changes in dermal fibroblasts and extracellular matrix components such as collagen, glycosaminoglycan, and elastin^6^. The most massive structure of extracellular matrix macromolecules is elastic fibers that are abundant in tissues such as skin, lungs, and arteries. The changes in elastin fibers with cutaneous aging are a complex biological phenomenon in different processes^7^. In skin damaged by prolonged sunlight exposure (photoaging), amorphous elastin-containing material is accumulated in the upper dermis and ultimately results in a lack of resilience^8–10^. On the contrary, elastin fibers naturally decrease with aging in skin protected from sunlight (intrinsic aging)^8,11^. Elastin reduction in skin causes wrinkles, sagging, and skin disorders^12,13^. Elastin turnover takes a long time, making it difficult to regenerate lost elastin^14,15^.

Lysosomal sialidase isozyme Neu1 constitutes an elastin receptor complex with elastin-binding protein (EBP), which has been identified as a sprite variant of β-galactosidase, and protective protein/cathepsin A on the cell surface. Cleavage of sialic acid from microfibrils by Neu1 facilitates elastic fiber assembly^16,17^. In the process of elucidating the function of sialidase in the dermis, it was found that sialidase activity in the skin significantly decreased with aging. Therefore, supplementation of sialidase by transdermal administration may restore the aging-related loss of elastic fibers.

For transdermal administration, an optimal selection of penetration enhancers is required for effective and noninvasive transdermal absorption because the structured lipids of the stratum corneum constitute a significant permeation barrier of the skin^18–20^. A large number of penetration enhancers including surfactants, fatty acids, and other solvents have been reported^21^. Recently, ionic liquids and deep eutectic solvents have been recognized as a new class of penetration enhancers. Ionic liquids are known as molten salts composed of cations and anions^22^. Deep eutectic solvents are widely acknowledged as a new class of ionic liquid analogs because they share many characteristics and properties with ionic liquids. Choline and geranate (CAGE) is one of the deep eutectic solvents^23^ and has been reported to show efficient transdermal delivery of various types of drugs from low molecular weight compounds^23^ to large molecular weight compounds such as peptides and proteins without any irritation to the skin^24,25^. Thus, it is thought that CAGE would enable delivery of sialidase to a deep site of the skin noninvasively.

In the present study, we first examined the distribution of sialidase activity in the skin and the changes in sialidase activity with aging. Based on this information, we investigated the effect of transdermal sialidase delivery with CAGE on cutaneous elastin. Here, we also suggest the role of the sialidase isozyme Neu2 in elastin expression.

## Results

### Change in sialidase activity in cutaneous tissue with aging

**Figure 1:**
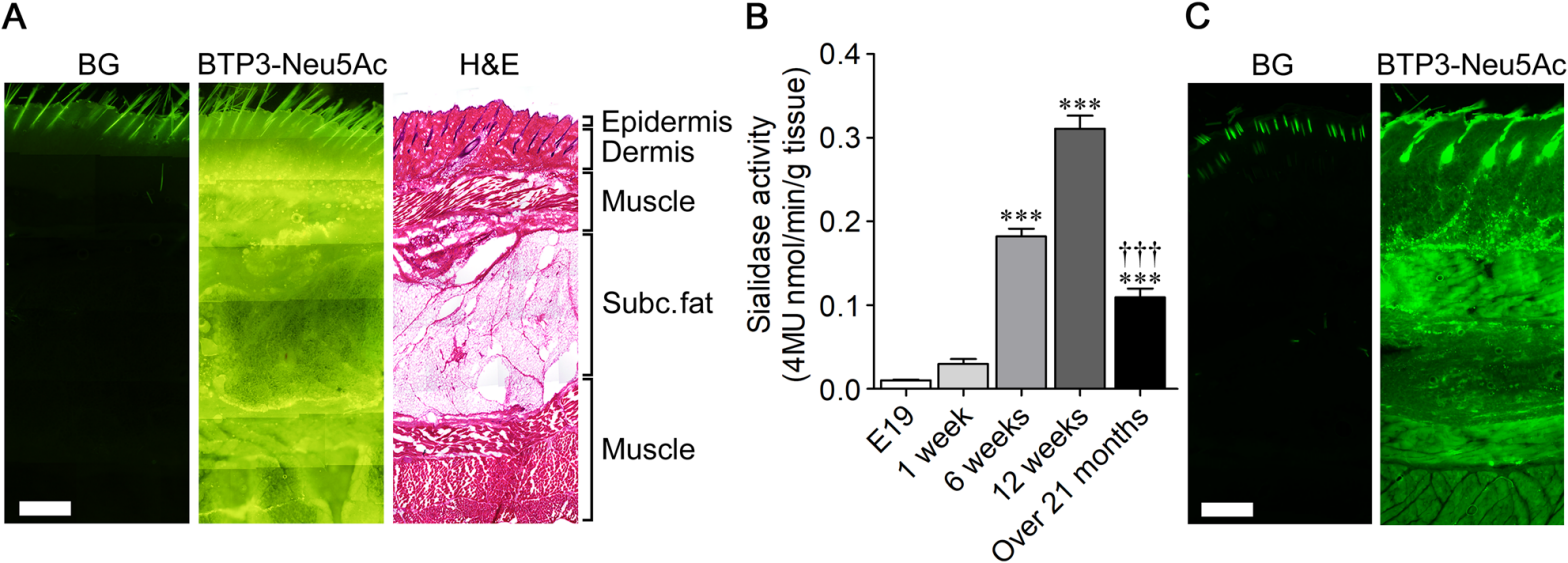
Decreases in sialidase activity in the rat skin with aging. (**A**) Rat tissues of the lateral abdomen were stained with 1 mM BTP3-Neu5Ac (middle column) at pH 7.3 and hematoxylin–eosin (H&E, right column). Background level (BG) for staining with BTP3-Neu5Ac is shown in the left column. Subc.fat: subcutaneous fat. (**B**) Change in sialidase activity in the skin with aging. *n* = 4–8. ***P < 0.001 vs. E19; ^†††^*P* < 0.001 vs. 12 weeks old (one-way ANOVA with Bonferroni's multiple comparison test). (**C**) Rat tissues of the lateral abdomen were stained with 1 mM BTP3-Neu5Ac at pH4.6. Scale bars, 500 μm.

To investigate the distribution of sialidase activity in the skin, lateral abdomen tissues of rats at 12 weeks of age were stained with 100 µM BTP3-Neu5Ac. A lower layer of the dermis and muscle identified by hematoxylin–eosin staining showed intense sialidase activity at pH7.3 compared to that in subcutaneous fat (Fig. 1A). The hair showed intense background fluorescence under the conditions of BTP3 fluorescence observation (ex/em, 372 nm/526 nm). We also investigated the change of sialidase activity in the skin with aging. Sialidase activity was increased from embryonic day (E) 19 to 12 weeks of age. The sialidase activity in rat skin over 21 months of age was significantly lower than that in rat skin at 12 weeks of age. (*F*_4,19_ = 241.0, *p* < 0.0001) (Fig. 1B). The distribution of sialidase activity at pH 4.6 was not significantly different from that at pH 7.3 (Fig. 1C).

### Increase in cutaneous elastin by transdermal sialidase delivery

**Figure 2:**
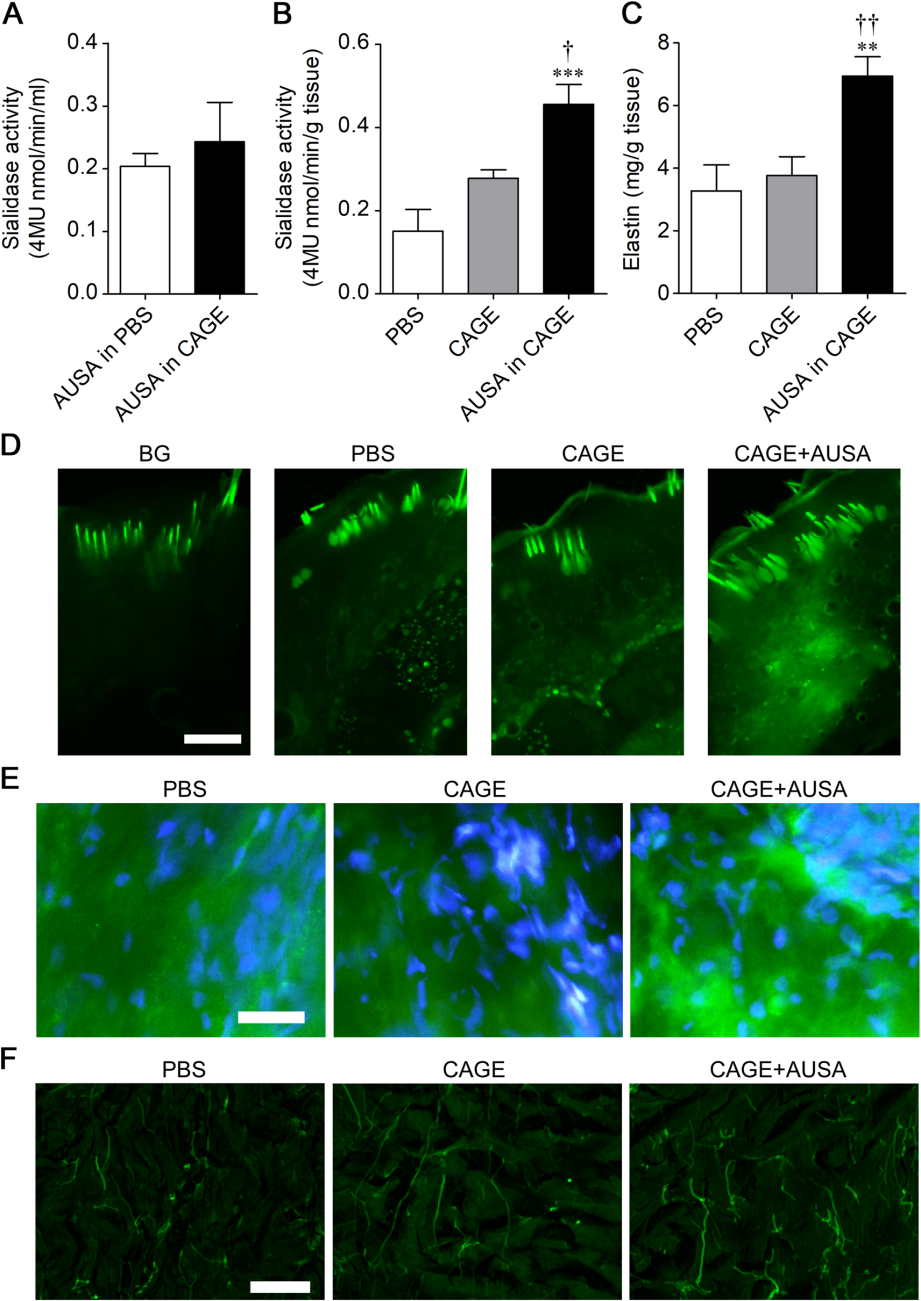
Enhancement of elastin expression in the rat skin by sialidase. (**A**) Enzyme activity of AUSA was measured with 4MU-Neu5Ac at 28 days after dissolving AUSA in CAGE. (**B**,**C**) Sialidase activity (*n* = 5–8, **B**) and elastin level (*n* = 7–8, **C**) in the rat lateral abdomen skin were measured after applying PBS, CAGE, or AUSA in CAGE on the skin twice a day for 5 days. **P < 0.01, ***P < 0.001 vs. PBS; ^†^*P* < 0.05, ^††^*P* < 0.01 vs. CAGE (one-way ANOVA with Bonferroni's multiple comparison test). (**D**) Sialidase activity imaging with BTP3-Neu5Ac (pH 7.3) in the rat lateral abdomen skin after applying PBS, CAGE, or AUSA in CAGE on the skin. Scale bar, 500 μm. (**E**) Immunohistochemical staining of elastin in extension sections of the dermis. Elastin: green, DAPI: blue. Scale bar, 300 μm. (**E**) Autofluorescence in the dermis. Scale bar, 50 μm.

It has been reported that the endogenous sialidase isozyme Neu1 is involved in elastin production. Elastin production may be restored by supplementing the sialidase that has decreased with aging. Here, we investigated the effect of transdermal administration of exogenous sialidase on the amount of elastin in the skin. CAGE was used for transdermal delivery of sialidase from *Arthrobacter ureafaciens* (AUSA). To confirm that sialidase activity is maintained in CAGE, the enzyme activity of AUSA was measured with 4-methylumbelliferyl-*α*-_D_-*N*-acetylneuraminic acid (4MU-Neu5Ac) at 28 days after dissolving AUSA in CAGE. The sialidase activity was not significantly altered by CAGE (*p* = 0.5882) (Fig. 2A).

Next, AUSA was transdermally delivered by using CAGE. Sialidase activity in the rat skin was increased by applying AUSA with CAGE on the skin twice a day for 5 days (*F*_2,19_ = 13.05, *p* = 0.0003) (Fig. 2B). We also measured the total amount of elastin, including tropoelastin, in the rat skin after solubilizing insoluble elastin with oxalic acid. The level of elastin was increased by applying AUSA with CAGE (*F*_2,17_ = 9.352, *p* = 0.0018) (Fig. 2C). Imaging with BTP3-Neu5Ac showed intense sialidase activity in the dermis after transdermal administration of AUSA with CAGE (Fig. 2D). In immunohistochemical staining in extension sections and observation of elastin autofluorescence, high expression of elastin was observed in the dermis after AUSA administration with CAGE (Fig. 2E,F).

### Increase in cutaneous elastin in senescence-accelerated mice by transdermal sialidase delivery

Senescence-accelerated mice (SAM) are characterized by an accelerated senescence process, short lifespan, and early onset and rapid progression of age-associated pathological phenotypes similar to human geriatric disorders^26–28^. Nine strains of senescence-prone (SAMP) mice and 3 strains of senescence-resistant mice have been established by selective inbreeding of the AKR/J strain^27^.

The SAMP1 strain is characterized by senescent amyloidosis and immunodeficiency. At 12 to 18 months of age, SAMP1 mice exhibit increases in elastic fibers and epidermal thickness that develop into overt elastosis, the characteristics of which are similar to those of human photoaging^29^. SAMP8 strain is characterized by Alzheimer’s disease-like neurodegenerative and cognitive deficit and a circadian rhythm disorder^30,31^. SAMP8 mice exhibit precocious aging similar to that of aged wild-type mice^32^.

**Figure 3:**
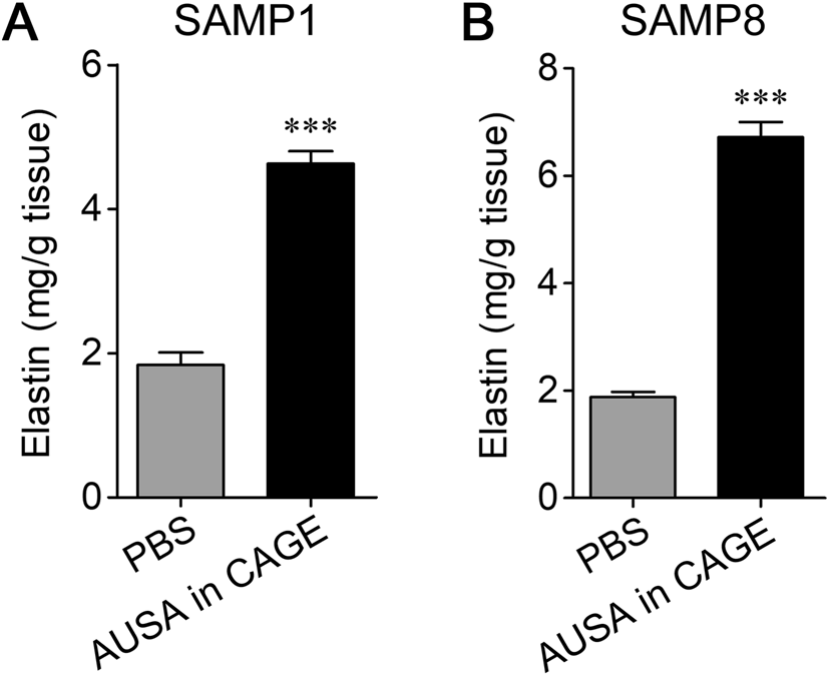
Enhancement of elastin expression by sialidase in SAMP1 and SAMP8. Elastin levels in the skin of SAMP1 (**A**) and SAMP8 (**B**) were measured after applying AUSA in CAGE or PBS on the skin twice a day for 5 days (*n* = 6 in each). ***P < 0.001 (unpaired *t*-test).

In this study, we investigated the effect of AUSA on the amount of elastin by using SAMP1 and SAMP8 strains. After transdermal administration of AUSA with CAGE twice a day for 5 days, the elastin level was significantly increased in SAMP1 and SAMP8 mice (SAMP1, *p* < 0.0001; SAMP8, *p* < 0.0001) (Fig. 3A,B).

### Expression of sialidase isozymes in cutaneous tissues

**Figure 4:**
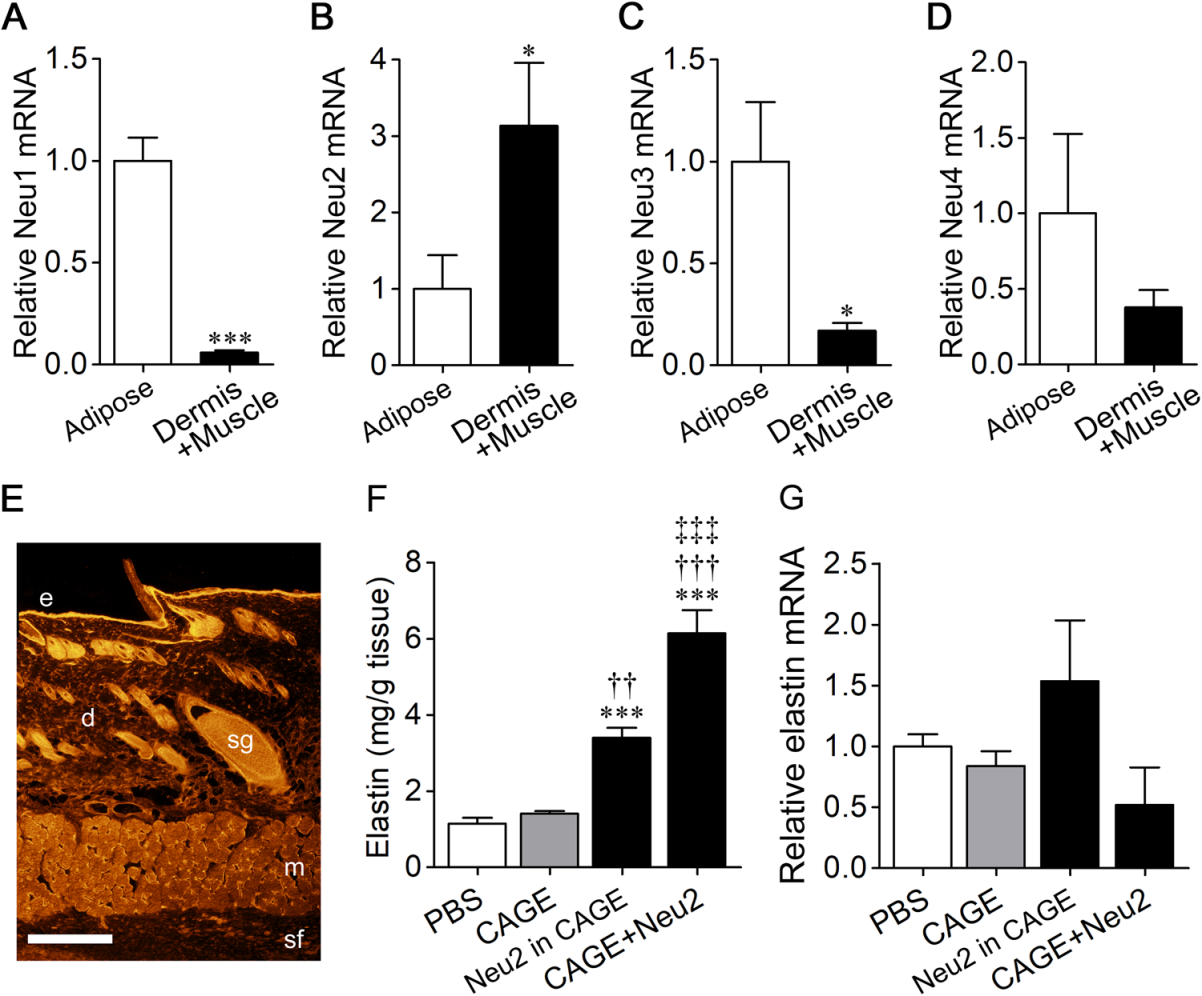
Enhancement of elastin expression by mammalian sialidase Neu2. (**A**–**D**) Expression of various sialidase isozyme mRNAs in adipose tissue (*n* = 6 in each) and tissues including the dermis and muscle (*n* = 5 in each). *P < 0.05, ***P < 0.001 (unpaired *t*-test). (**E**) Neu2 expression in lateral abdomen tissue was determined by immunohistochemical staining. e, epithelium; d, dermis; m, muscle; sf, subcutaneous fat; sg, sebaceous glands. Scale bar, 1 mm. (**F**,**G**) Elastin (*n* = 8 in each) and elastin mRNA levels (*n* = 6–7) in the rat skin were measured after applying PBS, CAGE or Neu2 in CAGE or after applying Neu2 first and then CAGE twice a day for 5 days. ***P < 0.001 vs. PBS; ^††^*P* < 0.01, ^†††^*P* < 0.001 vs. CAGE, ^‡‡‡^*P* < 0.001 vs. Neu2 in CAGE (one-way ANOVA with Bonferroni's multiple comparison test).

Mammalian sialidase has four isozymes: Neu1, Neu2, Neu3, and Neu4. The sialidase isozymes have different characteristics in terms of optimum pH, substrate specificity and subcellular localization^5^. Since intense sialidase activity was observed in the dermis and muscle, we investigated the expression of sialidase isozymes in rat subcutaneous tissue. The expression level of Neu2 mRNA was remarkably higher in tissues including the dermis and muscle than in adipose tissue (*p* = 0.0398) (Fig. 4A–D). On the other hand, the expression levels of Neu1, Neu3, and Neu4 were higher in adipose tissue than in tissues including the dermis and muscle (Neu1, *p* = 0.0004; Neu3, *p* = 0.0376; Neu4, *p* = 0.3002). Immunohistochemical staining of Neu2 in lateral abdomen tissue showed that Neu2 was expressed abundantly in dermis and muscle compared with subcutaneous fat (Fig. 4E).

### Increase in cutaneous elastin by transdermal delivery of Neu2

Although it is known that Neu1 increases elastin production, the effect of Neu2 on elastin production is unknown. Therefore, the effect of Neu2 on the amount of elastin in the skin was examined by using rat Neu2 expressed in C6 rat glioma cells. The enzyme activity of Neu2 measured with 4MU-Neu5Ac in phosphate-buffered saline (PBS, pH 7.3) was 21.3 nmol/min/ml. The Neu2 solution was mixed with CAGE at a volume ratio of 3:7 (Neu2: CAGE) and then applied on the rat skin. Alternatively, Neu2 (18 µL) was applied on the skin first, and then CAGE (42 µL) was applied. The application of Neu2 was performed twice a day for 5 days, resulting in elevated elastin levels in the skin by both methods (*F*_3,28_ = 45.75, *p* < 0.0001) (Fig. 4F). The elastin levels were much higher with application of Neu2 first and then CAGE than by application of the mixed solution of Neu2 and CAGE. Elastin mRNA levels in the skin were not significantly affected by applying Neu2 (*F*_3,24_ = 1.75) (Fig. 4G).

## Discussion

In the present study, we obtained basic knowledge about sialidase in the skin such as the distribution of sialidase enzyme activity, the expressed isozymes, and changes in expression with aging. When tissue of the rat lateral abdomen was stained with BTP3-Neu5Ac at pH 7.3, intense sialidase activity was detected in the dermis and muscle compared to that in adipose tissue. BTP3-Neu5Ac is preferentially hydrolyzed by sialidase isozymes Neu2 and Neu4 and is weakly hydrolyzed by Neu1 and Neu3 at pH 7.3^33^. In contrast to Neu1, Neu3 and Neu4, the expression of Neu2 mRNA in the dermis and muscle was abundant compared with that in adipose tissue. Thus, intense sialidase activity in the dermis and muscle at pH7.3 was presumed to be due to Neu2. In addition, the distribution of sialidase activity at pH 4.6 was not significantly different from that at pH 7.3. At pH 4.6, BTP3-Neu5Ac is efficiently hydrolyzed by Neu1 and Neu3, but also by Neu2 and Neu4^33^. Neu2 may be mainly contributed to the imaging of sialidase activity in the skin even at pH 4.6.

Skin aging is attributed to qualitative and quantitative changes such as changes in collagen, elastin, and glycosaminoglycans in the dermis^6^. Reduction of cutaneous elastin causes loss of skin elasticity and has an impact on the health of skin. Neu1 is involved in the facilitation of elastic fiber assembly^16^. Cleavage of sialic acid from the glycoprotein microfibrils by Neu1 allows EBP to bind to the microfibrils. Then tropoelastin, a precursor protein to elastin, is released from EBP, assembled around the microfibrils, and cross-linked between the molecules to form mature elastin fibers^17^. In the present study, we found that sialidase activity measured with 4MU-Neu5Ac at pH 7.3 was decreased by aging. The decrease in elastin associated with aging may be related to the reduction in sialidase activity.

Human skin has a large variability in drug permeability depending on the site of skin used, age and race. Thus, rodents and porcine skin have been widely used for in vitro skin permeation studies in preclinical studies for drug development as a replacement for human skin. Among rodents, rat skin is the most structurally similar to human skin and is frequently used for in vivo pharmacokinetics, pharmacological, and toxicological studies^34^. Some previous reports showed that the permeation rates of drugs in rat skin correlates well with values in human skin, although permeability of highly polar compounds through rat skin tends to be higher than that through human skin^34,35^. In this paper, only rodent skin was used. However, evaluation using a human skin model is necessary for future application to humans.

We tried to promote elastin production by transdermal sialidase administration by using CAGE. CAGE is useful for transdermal delivery of proteins with stability, simplicity, and little variation. Sialidase activity of AUSA is maintained in CAGE at the same level as in PBS. Sialidase activity of AUSA is also maintained in the rat skin after the transdermal delivery of AUSA with CAGE. These results suggest that CAGE can deliver the enzyme subcutaneously while maintaining the enzymatic activity. Transdermal delivery of AUSA with CAGE increases the amount of elastin in the skin. Neu1 is involved not only in the facilitation of elastic fiber assembly but also in modulation of the signaling of elastin-derived peptides for elastin degradation^16^. Since the transdermal sialidase administration increased the amount of elastin, exogenous sialidase may facilitate elastic fiber assembly rather than disassembly.

Neu2 regulates myoblast and neuronal differentiation and apoptosis signaling^36,37^. Transdermal administration of Neu2 with CAGE increased the amount of elastin in the skin. Thus, it is thought that not only Neu1 but also Neu2 is involved in elastin production. Elastin mRNA levels in the skin were not significantly affected by applying Neu2. Thus, the increase in elastin by sialidase is due to the facilitation of elastic fiber assembly by sialic acid desorption from microfibrils. Based on the observation of elastin, severe dermal elastosis as occurs in photoaged skin was not observed in Neu2-treated skin^38^. However, more detailed histochemical staining will be needed to rigorously check that sialidase does not induce dermal elastosis.

Neu1 is a lysosomal enzyme and has an optimal pH of 4.4–4.6. On the other hand, Neu2 is located mainly in the cytoplasm but also in plasma membranes^39^. Neu2 hydrolyzes glycoproteins, oligosaccharides and gangliosides and has an optimum pH of 6.0–6.5^37^. Since Neu2 can act at extracellular neutral pH, exogenously administered Neu2 may efficiently release sialic acid from microfibrils in the extracellular space. Besides, application of Neu2 first and then CAGE more efficiently promoted elastin production than did mixing the Neu2 solution with CAGE. In preparing the Neu2 solution in CAGE, there is water contamination because Neu2 suspended in PBS was mixed with CAGE. Water attenuates the effect of the ionic liquid. On the other hand, when Neu2 was applied to the skin alone, the water is removed at this stage. It is presumed that the subsequent application of CAGE allows for efficient penetration of Neu2. It is expected that the enzyme can be delivered subcutaneously more efficiently by further optimizing the administration method with CAGE.

Elastin is decreased with intrinsic aging in the skin^8,11^. In addition to aging, elastin was also shown to be reduced by dermatochalasis^40^, pseudoxanthoma elasticum^41^, Williams-Beuren syndrome^42^, and skin disorders due to obesity^43^, which deteriorate the skin environment. An increase in subcutaneous adipose tissue leads to increased degradation of dermal elastic fibers. Transdermal administration of AUSA with CAGE also increased the amount of skin elastin in the SAMP1 and SAMP8 strains of senescence-accelerated mice. Although elastin turnover takes a long time^14,15^, the skin environment would be improved by recovering reductant elastin by transdermal sialidase administration.

In summary, we showed that the dermis of the skin has intense sialidase activity mainly due to Neu2 and that the activity decreases with aging. CAGE was confirmed to be useful for transdermal delivery of sialidase while maintaining its enzymatic activity. CAGE may be useful for transdermal administration of various enzymes as well as sialidase. We also showed that cutaneous elastin was increased by transdermal delivery of sialidase with CAGE. Therefore, transdermal administration of sialidase is expected to be useful for improvement of wrinkles and skin disorders due to loss of elastin.

## Methods

### Materials and animals

Male Wistar rats (E19—12 months of age or older), male SAMP1 (24 weeks of age), and male SAMP8 (24 weeks of age) were purchased from Japan SLC (Shizuoka, Japan). The rats and mice were housed under standard laboratory conditions (23 °C ± 1 °C, 55% ± 5% humidity) and had access to tap water and diet ad libitum. The lights were automatically turned on at 8:00 and turned off at 20:00. All experiments were carried out in compliance with the guidelines established by the University of Shizuoka for the care and use of laboratory animals, and the ARRIVE guidelines. The protocols were pre-approved by the Animal Ethics Committee of the University of Shizuoka.

BTP3-Neu5Ac was synthesized according to the procedure described previously^3^. Geranic acid (85%), chorine bicarbonate (~ 80% in water), and dimethyl sulfoxide-d6 (DMSO-d6, 99.5 atom %D) were purchased from Merck KGaA (Darmstadt, Germany).

### Sialidase activity imaging

Rats (12 weeks of age) were anesthetized with a mixture of butorphanol tartrate (Meiji Seika Pharma Co., Ltd., Tokyo, Japan; 2.5 mg/kg body weight), medetomidine hydrochloride (Fujifilm Wako Pure Chemical Co., Osaka, Japan; 0.375 mg/kg body weight) and midazolam (Fujifilm Wako Pure Chemical Co., 2 mg/kg body weight) by intraperitoneal injection. After transcardial perfusion with 200 ml of phosphate-buffered saline (PBS), lateral abdomen tissues including skin, muscle, and subcutaneous fat were quickly harvested and embedded in Tissue-Tek OCT compound (Sakura Finetek, Tokyo, Japan). After being frozen, the tissues were cut at − 20 °C using a cryotome into 300-μm-thick sections for staining shown in Fig. 1A,C and 100-μm-thick sections for staining shown in Fig. 2D.

Sialidase activity imaging was performed according to the procedure described previously^44^. Briefly, the sections were stained with BTP3-Neu5Ac (300-μm-thick sections: 1 mM for 60 min, 100-μm-thick sections: 300 μM for 30 min) in PBS or 100 mM sodium acetate buffer (pH4.6) at 27 °C. After washing with PBS, fluorescence was observed using a fluorescence microscope (Olympus IX71 or Keyence BZ-X710) with a filter set (ex/em: BP330-385/BA510IF for IX71 and BP340-380/BA500-550 for BZ-X710). In all observations with the fluorescence microscope, the gain of the microscope camera was set so as not to detect background fluorescence in a non-stained skin section. After obtaining pictures, the slices were fixed with 4% paraformaldehyde in PBS and then stained with hematoxylin–eosin. If necessary, the images were tiled together using Photoshop CS4 (Adobe Systems, San Jose, CA).

### Measurement of sialidase activity

The rat skin was homogenized with sucrose (0.32 M) at 4 °C. Homogenates were transferred to a 96-well black microplate (Corning, NY, USA) and then incubated in PBS containing 4MU-Neu5Ac (10 µM, Nacalai Tesque, Kyoto, Japan) for 60 min at 27 °C. After the addition of sodium carbonate buffer (500 mM, pH 10.7), fluorescent intensities of 4-methylumbelliferone were measured using a microplate reader (ex/em, 355 nm/460 nm; Infinite M200, Tecan, Männedorf, Switzerland).

### Preparation of CAGE

CAGE was prepared using a previously established method^23^. Two equivalents of recrystallized geranic acid (85%, 5.070 g) were added to one equivalent of choline bicarbonate (80 wt% solution, 3.109 g) in a 100 mL eggplant flask. The mixture was stirred at room temperature until no more CO_2_ evolved. The solvent was removed by rotary evaporation at 60 °C for 20 min, and the product was dried in a vacuum oven for 48 h at 60 °C to obtain CAGE (6.583 g). NMR assignments (collected using a JEOL ECX500, Tokyo, Japan) were in good agreement with those in a previous study^23^.

### Measurement of the stability of sialidase in CAGE

Lyophilized sialidase from *Arthrobacter ureafaciens* (AUSA, Nacalai Tesque, Kyoto, Japan) was dissolved in CAGE or PBS at 10 mU/ml and then left at 27 °C for 28 days. Then the enzyme activity of sialidase was measured by mixing AUSA solution (10 μl), 4MU-Neu5Ac in PBS (100 μM, 40 μl), and PBS (50 μl).

### Transdermal sialidase administration

Ionic liquid solutions of sialidase were prepared by dissolving lyophilized sialidase in CAGE. After shaving the hair, the solution of AUSA (1 U/ml) in CAGE, CAGE, or PBS was applied on the lateral abdomen skin of rats (12 weeks of age) and SAMP1 and SAMP8 twice a day for 5 days. Dermis tissues were quickly harvested and homogenized with sucrose (0.32 M) at 4 °C. Sialidase activity in the homogenate was measured with 4MU-Neu5Ac (40 µM). The level of elastin extracted from the dermis (20 mg) was measured with a quantitative dye, 5,10,15,20-tetraphenyl-21,23-porphine tetra-sulfonate, using a Fastin Elastin Assay Kit (Biocolor, Northern Ireland, UK) according to the manufacturer’s instructions.

### Immunohistochemical staining

For elastin staining, extension sections of the dermis were fixed with 4% paraformaldehyde in PBS. After blocking with 2% goat serum in tris-buffered saline with Tween 20, the sections were stained with rabbit anti-elastin polyclonal IgG (Bioss Antibodies, Woburn, MA) and FITC-conjugated goat anti-rabbit IgG (Jackson ImmunoResearch Laboratories, West Grove, PA). The anti-elastin polyclonal IgG was produced using a synthetic peptide derived from human elastin, and has cross-reactivity to human, mouse and rat elastin. Counterstaining was performed using DAPI (1 µg/ml).

For Neu2 staining, lateral abdomen tissues were fixed with 4% paraformaldehyde in PBS. After blocking with 2% goat serum in tris-buffered saline with Tween 20, the sections were stained with rabbit anti-Neu2 polyclonal IgG (Rockland, Gilbertsville, PA) and HiLyte Fluor 555-conjugated goat anti-rabbit IgG (Anaspec, Fremont, CA).

Images were acquired by using a fluorescence microscope BZ-X710 with a filter set (ex/em: BP450-490/BA500-550 for FITC and BP340-380/BA435-485 for DAPI) and by using a fluorescence microscope IX71 (Olympus, Tokyo, Japan) with a filter set (ex/em: BP530-550/BA575IF for HiLyte Fluor 555). The background level of fluorescence was determined by sections stained with only the secondary antibody. All imaging was performed at least twice in different rats, and reproducibility was confirmed.

### Elastin autofluorescence^45^

Unstained sections of the dermis were fixed with 4% paraformaldehyde in PBS. Autofluorescence was observed using a fluorescence microscope BZ-X810 (Keyence) with a filter set (ex/em: BP450-490/BA500-550).

### Real-time quantitative reverse transcription-polymerase chain reaction (real-time RT-PCR)

The procedure for real-time RT-PCR was described previously^44^. Briefly, total RNA was isolated from rat tissues (at 12 weeks of age) such as skin and adipose in the lateral abdomen by using the guanidinium phenol reagent (TRIzol reagent, Life Technologies) according to the manufacturer’s instructions. The expression level of mRNA was evaluated using a thermal cycler system (Thermal Cycler Dice Real-Time System Lite, TaKaRa Bio), a One-Step SYBR PrimeScript PLUS RT-PCR kit (Perfect Real Time, TaKaRa Bio) and primer pairs [5′-CCCATCCCGAGTACCGAGT-3′ and 5′-CCCGGCCACAACTGGAC-3′ for Neu1, 5′-GAGCCACCAACCATGTCAAG-3′ and 5′-AAGGGACATGGATTCATGGAG-3′ for Neu2, 5-CGGAGCTGGTGAGCTGAG-3′ and 5′-CCTGCTGGAACAGAGTGCTG-3′ for Neu3, 5′-TCTGGAGAGTGCCAACTGGC-3′ and 5′-AAGGAAGTGCCTTCATCAGCAC-3′ for Neu4, 5′-GCTTAGGAGTCTCAACAGGTGC-3′ and 5′-CGGAACCTTGGCCTTGACTC-3′ for elastin, and 5′-TGAACGGATTTGGCCGTATCGG-3′ and 5′-TCAATGAAGGGGTCGTTGATGG-3′ for glyceraldehyde-3-phosphate dehydrogenase (GAPDH)]. GAPDH mRNA was used as an internal standard to normalize sample variation.

### Transdermal administration of Neu2

The previously established C6 rat glioma cells stably expressing C-terminal Myc-tagged rat Neu2 were cultured for 48 h in a humidified incubator with 5% CO_2_ at 37 °C in DMEM supplemented with 10% fetal bovine serum^33^. Neu2 secreted extracellularly was collected from the culture supernatant (4 ml) by ultrafiltration with the Amicon Ultra filter unit (Millipore Co., Darmstadt, Germany) at 4000 × *g* for 20 min. After washing with PBS, Neu2 activity was measured with 4MU-Neu5Ac in PBS (40 µM, pH 7.3). Neu2 (21.3 nmol/min/ml) was mixed with CAGE at a ratio of 3:7 (Neu2 : CAGE). After shaving, 60 µL of a mixture of Neu2 and CAGE, CAGE, or PBS was applied on the rat lateral abdomen skin (at 12 weeks of age). Alternatively, 42 µL of CAGE was applied on the skin after applying 18 µL of Neu2. The application was performed twice a day for 5 days. The level of elastin in the dermis was measured in the same manner as described above.

### Statistical analysis

Statistical significance was assessed by one-way ANOVA with Bonferroni’s multiple comparison test and two-tailed unpaired *t*-test. If the variances between the two groups were different, the two-tailed unpaired *t*-test with Welch’s correction was used. *F* value in one-way ANOVA was denoted with the degrees of freedom for the numerator and for the denominator.

## Abbreviations

AUSA: Sialidase from *Arthrobacter ureafaciens*
BTP3-Neu5Ac: Benzothiazolylphenol-based sialic acid derivative type 3
CAGE: Choline and geranate
DMSO-d6: Dimethyl sulfoxide-d6
EBP: Elastin-binding protein
PBS: Phosphate-buffered saline
SAM: Senescence-accelerated mouse
SAMP: Senescence-prone
4MU-Neu5Ac: 4-Methylumbelliferyl-*α*-_D_-*N*-acetylneuraminic acid
